# Genome-Wide Analysis of microRNAs Identifies the Lipid Metabolism Pathway to Be a Defining Factor in Adipose Tissue From Different Sheep

**DOI:** 10.3389/fvets.2022.938311

**Published:** 2022-07-08

**Authors:** Tian-Yi Liu, Hui Feng, Salsabeel Yousuf, Ling-Li Xie, Xiang-Yang Miao

**Affiliations:** State Key Laboratory of Animal Nutrition, Institute of Animal Sciences, Chinese Academy of Agricultural Sciences, Beijing, China

**Keywords:** sheep, subcutaneous fat, microRNAs, high-throughput sequencing, fat deposition

## Abstract

microRNAs are a class of important non-coding RNAs, which can participate in the regulation of biological processes. In recent years, miRNA has been widely studied not only in humans and mice, but also in animal husbandry. However, compared with other livestock and poultry breeds, the study of miRNA in subcutaneous adipose tissue of sheep is not comprehensive. Transcriptome analysis of miRNAs in subcutaneous adipose tissue of Duolang sheep, and Small Tail Han sheep was performed using RNA-Seq technology. Differentially expressed miRNAs were screened between different breeds. Target genes were predicted, and then the joint analysis of candidate genes were conducted based on Gene Ontology (GO) and Kyoto Encyclopedia of Genes and Genomes (KEGG) enrichment. Finally, the RNA-Seq data were verified by real-time quantitative polymerase chain reaction (qRT-PCR). Herein, we identified 38 differentially expressed miRNAs (9 novel miRNAs and 29 known miRNAs). In addition, a total of 854 target genes were predicted by miRanda software. GO and KEGG pathway analysis demonstrated that regulation of lipolysis in adipocytes plays a key role in the deposition of subcutaneous adipose tissue in Duolang sheep and Small Tail Han sheep. The miRNAs might regulate fat deposits by regulating genes involved in regulation of lipolysis in adipocytes. Specifically, NC_ 040278.1_ 37602, oar-mir-493-3p, NC_ 040278.1_ 37521 and NC_ 040255.1_ 11627 might target PTGS2, AKT2, AKT3, and PIK3CA, respectively, and then play critical regulatory role. In conclusion, all the results provide a good idea for further revealing the mechanism of subcutaneous adipose tissue deposition and improving the meat production performance of sheep, and lay a foundation for promoting the development of animal husbandry.

## Introduction

miRNA is a kind of short-chain endogenous non-coding RNA with a length of 18–25 nt, which is highly conservative and widely distributed in animals, plants and viruses (1, 2). With the discovery of lin-4 and let-7 in Caenorhabditis elegans (3, 4), the research of miRNA has gradually entered people's vision. Although miRNAs cannot encode proteins, they still control various biological processes and are key regulators of development and cellular homeostasis (5). In many biological processes, miRNAs regulate gene expression by recognizing homologous sequences and interfering with transcriptional, translational or epigenetic processes (6). In the past few years, the research of miRNA in cancer has exploded (7), including diseases such as hepatocellular carcinoma (8), breast cancer (9), and gastric cancer (10). Not only that, miRNA in type 2 diabetes (11), hypertension (12) and atherosclerosis (13) is also very common. Obesity has become a prevalent health problem as it is closely associated with many diseases.

Obesity is due to the proliferation, differentiation or enlargement of adipocytes, and miRNAs have both promoting and inhibiting effects on adipocyte differentiation (14). LPL is a direct target gene of miR-152 during preadipocyte differentiation. MiR-152 can inhibit the proliferation of 3T3-L1 preadipocytes and promote the differentiation of 3T3-L1 preadipocytes by negatively regulating LPL (15). The study found that miR-244 regulates the adipogenic differentiation of bovine preadipocytes by targeting LPL. When miR-224 was overexpressed, the mRNAs expression levels of the adipogenesis-related markers PPARγ, FASN, C/EBPα, C/EBPβ and PLIN1 decreased, whereas the opposite effect was produced when miR-224 was inhibited (16). The regulation of lipid metabolism by miRNA is a complex network regulation system, which mainly affects lipid metabolism by regulating genes related to lipid synthesis, oxidation, and transport (17, 18). For example, miR-33, as one of the widely studied miRNAs, is involved in the regulation of multiple lipid metabolism links. The expression of genes related to fatty acid β-oxidation can be inhibited, and the biosynthesis of high-density lipoprotein and the outflow of cholesterol can also be inhibited (19, 20). As the first miRNA found to be involved in the regulation of lipid metabolism, miR-122 has been deeply studied. MiR-122 is most abundant in the liver and plays an important role in mainly regulating lipid synthesis and oxidation processes, especially the accumulation of triglycerides. MiR-122 plays an important regulatory role in the translation of YY1 and FXR. Mir-122 upregulates FXR expression by targeting the 3'UTR of YY1 mRNA upstream, suppressing triglyceride levels in hepatocytes, and FXR is also involved in the regulation of lipid metabolism disorders and insulin resistance (21). Another study showed that the expression of miR-122 was inhibited by binding to the 3'UTR of Sirt1 in non-alcoholic fatty liver disease, and down-regulation of miR-122 inhibited adipogenesis genes, but activated the AMPK signaling pathway, further inhibiting hepatic lipogenesis and triglyceride secretion (22). Studies have found that miR-122 can also promote cholesterol synthesis (23, 24). The above studies show that miRNA plays a key regulatory role in the process of fat deposition.

As one of the main meat livestock and poultry resources in the world, sheep has warm meat, high protein content and low fat and cholesterol levels. Compared with pork, the meat quality is more delicate, and the content of essential amino acids is also higher than that of pigs, chickens and cattle (25, 26). Adipose tissue is an important factor affecting meat quality, mainly including the effects on sensory, flavor and tenderness (27, 28). Moreover, the back-fat of sheep is an important indicators of meat yield and hot carcass composition (29). And subcutaneous adipose tissue accounts for the highest proportion of total fat content in animals (30), which has the function of protecting animals and storing energy. Duolang sheep and Small Tail Han sheep belong to high-quality local breeds in China, and there are differences in fat deposition between the two breeds. Duolang sheep have large fat buttocks, strong meat production capacity, fresh and juicy meat, and belongs to both meat and fat sheep, and Duolang sheep also have the characteristics of rapid growth and development. Small Tail Han sheep are short and thin-tailed sheep, with strong environmental adaptability and reproductive ability, fast growth rate and stable genetic performance, but the meat body size is not obvious and the carcass meat production rate is low. At present, living standards have been greatly improved, and people's requirements for meat quality and quantity have also increased. Therefore, studying the molecular mechanism of sheep fat deposition can improve the quality of meat products to meet consumer demand. At the same time, adipose tissue is an important organ of energy metabolism, and excessive fat deposition can lead to obesity and the occurrence of a series of metabolic syndromes (31). Therefore, studying the mechanism of fat deposition can not only improve meat quality and promote the development of animal husbandry, but also prevent or treat a series of diseases affecting human health caused by excessive fat deposition. We selected the subcutaneous adipose tissue of Duolang sheep and Small Tail Han sheep as experimental materials, analyzed the gene expression profile of subcutaneous adipose tissue by transcriptome sequencing and bioinformatics methods, screened and identified the key candidate genes related to lipid metabolism and adipogenic differentiation and explored the molecular mechanism related to fat deposition.

## Materials and Methods

### Sample Collection and RNA Isolation

All work in this study was approved by the Animal Welfare and Ethics Committee of Beijing Institute of Animal Sciences, Chinese Academy of Agricultural Sciences (No. IAS2019-82). In order to detect the expression profile of miRNAs in sheep subcutaneous fat, we collected subcutaneous adipose tissue samples from 3 adult female Duolang sheep (D-PF-1, 2, 3) and 3 adult female Small Tail Han sheep (X-PF-1, 2, 3). All sheep were raised in the same conditions with free to drink and eat under natural light, and their diet meets the current nutritional needs. They were in good physical condition, aged 2 years old and the mean bodyweight of sheep were 50 ± 3 kg. We collected subcutaneous adipose tissue samples located at the back fat according to the agricultural industry standard of the people's Republic of China (NY/T 3469-2019). In order to reduce the pain of animals, we first stunned them with electricity and then slaughtered them. The whole sampling process was controlled within 30 min. The collected samples were immediately frozen in liquid nitrogen and stored in an environment of −80°C before the experiment.

Total RNA was isolated from each adipose tissue using TRIzol reagent (Invitrogen, Invitrogen Life Technologies, Carlsbad, USA), and genomic DNA was removed using rDNase I RNase-free (TaKara). The RNA concentration and quality were then measured with a 2100 Bioanalyzer (Agilent Technologies, Santa Clara, CA, USA) and the ND-2000 (NanoDrop Technologies), RIN > 8 was good quality.

### Library Preparation and Sequencing

The RNA library was constructed using the TruSeqTM Small RNA sample prep Kit (Invitrogen) according to the instructions. The rRNA in the total RNA was first removed, the 3′ end adapter and the 5′ end adapter were, respectively, connected with the kit, and then the random primers were reversed to 1st cDNA. To enrich the library we performed 11-12 PCR cycles. The library was enriched and then purified (6% Novex TBE PAGE gel, 1.0 mm, 10 well) and quantified by TBS380 (Picogreen). Bridge PCR was performed on cBot to generate clusters. The library was sequenced with the Illumina NovaSeq 6000.

### Quality Control and Sequence Alignment

In order to improve the sequencing quality and get clean reads, introduced adapter sequences, low-quality reads, sequences with high N rate (N stands for indeterminate bases), and sequences that are too short will be removed. The clean reads were aligned with the reference genome using Bowtie (32) software. The reference genome GCF_016772045.1 was obtained in the NCBI database. StringTie (32) software was used to splicing the mapped reads.

### miRNA Identification and Structural Analysis

The reads aligned to the reference genome were aligned with the miRNA precursor and mature sequences in the miRBase V.22.1 (33) to obtain known miRNAs. The Rfam (34) was used to filter ncRNAs and repeats sequences such as ribosomal RNA (rRNA), transfer RNA (tRNA), small nuclear RNA (snRNA) and small nucleolar RNA (snoRNA), and at that time, the types and numbers of these sequences were counted. The sRNAs that cannot be aligned with Rfam and miRBase were aligned to the reference genome, and the surrounding sequences were intercepted using miRDeep2 (35) software for secondary structure prediction to identify new miRNAs. In the process of developing from precursor to mature miRNA, the Dicer restriction site was specific, which makes the first base of miRNA mature sequence strongly biased to U. In addition, some non-canonical editing sites exist in miRNAs, thereby altering target genes. And the MiRME (36) method was used to detect various mutation and editing sites of miRNA. Finally, based on the seed sequence, the identified known miRNAs and new miRNAs were subjected to miRNA family analysis.

### Differentially Expressed miRNAs

In order to facilitate the subsequent analysis of the differential expression among the samples, quantitative analysis was performed on the expression levels of the samples, respectively. RSEM (37) software was used for quantification, and the expression level of each miRNA was calculated according to the transcripts per million readsx (TPM) method (38). DESeq2 (39) is a statistical analysis based on negative binomial distribution, which can be used in experiments with biological replicates. Significant differently expressed miRNAs were extracted with *p*-value < 0.05 and |log_2_FC|≥1.

### miRNA Target Gene Prediction

Because miRNAs cannot encode proteins, they function through post-transcriptional regulation of their target genes. In animals, miRNA relies on the seed sequence (2–8 nt at the 5′ end) to closely bind to the 3′ non-coding region of the target gene, thereby inhibiting the translation of the target mRNA. In this study, the miRanda (40) software was used to predict its target genes, and the parameters were set as: Score ≥ 160 and Energy ≤ −20. The predicted target genes were visualized by the application software Cytoscape (41). All of the mRNA data was obtained from fat collected from the same animals that were used for the miRNA analysis at the same relative time (age) and using a similar procedure.

### GO and KEGG Enrichment Analysis of Differentially Expressed Genes

GO (42) can be used for functional enrichment analysis on the differentially expressed genes. The software Goatools was used to perform GO enrichment analysis on the differentially expressed genes, so as to obtain functional annotations of the genes, including three parts: biological process, molecular function and cellular components. The KEGG (43) pathway enrichment analysis was also performed on the genes, and the R software was used for the enrichment analysis of metabolic pathways and information processing pathways. Using Fisher's exact test and Benjamini and Hoceberg (BH) to correct *p*-value to obtain *p*-adjust, when *p*-adjust < 0.05, we considered significant enrichment.

### Quantitative Real-Time Polymerase Chain Reaction

Real-time fluorescent quantitative PCR (qRT-PCR) was used to verify the reliability of the sequencing results. Five differentially expressed miRNAs and eight differentially expressed mRNAs were randomly selected for verification. In total, 0.5 μg RNA was taken to synthesize cDNA template through GeneAmp^®^ PCR System 9700 (Applied Biosystems, USA). First, RNA, 4 × g DNA wiper Mix and nuclease free H_2_O were reacted in GeneAmp^®^ PCR System 9700 at 42°C for 2 min. Second, 5 × HiScript II Q RT SuperMix IIa were added and reacted at 50°C for 15 min, then for 5 s at 85°C. And then the reverse transcription reaction mix were dilute × 10 in nuclease free H_2_O and maintained at −20°C. The qRT-PCR analysis was conducted using LightCycler^®^ 480 II Real-time PCR Instrument (Roche, Swiss). U6 and ACTB were used as miRNA and mRNA internal references, respectively. Three biological replicates were employed for each gene. The relative expression levels of genes between samples were calculated using 2^−ΔΔCt^ method. Data obtained were analyzed using GraphPad Prism (V8.0.1). The student *t*-test (*p* < 0.05) was used for mean comparisons. All results were presented in bar charts with the means and their standard deviation (±SD). The sequences of the primers used are listed in Supplementary Table S1.

### Statistical Analysis

All the data were presented as means ± SD. When comparisons were made, a student's *t*-test was performed and *p*-value < 0.05 was considered as statistically significant.

## Results

### Evaluation of RNA-Sequencing Data

In order to study the mechanism of miRNAs in subcutaneous adipose tissue of Duolang sheep and Small Tail Han sheep, we constructed cDNA libraries of two breeds, and obtained the original data by high-throughput sequencing technology. We obtained a total of 118.41 M Raw Reads in 6 sheep subcutaneous adipose tissue samples, and the Raw Reads in each sample reached more than 18.32 M, the percentage of Q20 bases was more than 97%, and the percentage of Q30 bases was more than 93% (Table 1). After quality control, compared with the reference genome, the mapping rate of each sample ranges from 93.41 to 97.14%, indicating that the reference genome is fully annotated and there is no contamination in the experiment, which is a good foundation for subsequent data analysis (Table 2).

**Table 1:** Quality control statistics of sequencing data for each sample.

**Sample**	**Raw reads**	**Clean reads**	**Error rate (%)**	**Q20 (%)**	**Q30 (%)**	**GC content (%)**	**Useful reads (18–32 nt)**
D_PF_1	19,305,542	19,237,461	0.0239	98.5	95.16	45.08	18,716,909
D_PF_2	19,293,493	19,262,447	0.0238	98.57	95.3	43.86	19,040,661
D_PF_3	18,321,730	18,244,173	0.0239	98.54	95.15	44.95	17,764,605
X_PF_1	19,036,709	18,972,125	0.0253	97.76	94.08	44.79	18,542,581
X_PF_2	23,072,665	22,989,021	0.0244	98.22	94.73	44.2	22,450,480
X_PF_3	19,383,731	19,336,376	0.0257	97.53	93.73	45.16	18,945,034

**Table 2:** Reference genome comparison results for each sample.

**Sample**	**Total reads**	**Total mapped**	**Mapped reads (+)**	**Mapped reads (–)**
D_PF_1	18,716,909	17,572,239 (93.88%)	13,567,787	7,418,996
D_PF_2	19,040,661	18,495,698 (97.14%)	14,384,579	8,158,010
D_PF_3	17,764,605	16,903,298 (95.15%)	13,052,330	7,106,665
X_PF_1	18,542,581	17,787,015 (95.93%)	12,868,296	8,980,321
X_PF_2	22,450,480	21,147,342 (94.20%)	14,790,236	11,768,078
X_PF_3	18,945,034	17,696,401 (93.41%)	13,532,382	7,894,160

### miRNA Identification and Prediction

**Figure 1:**
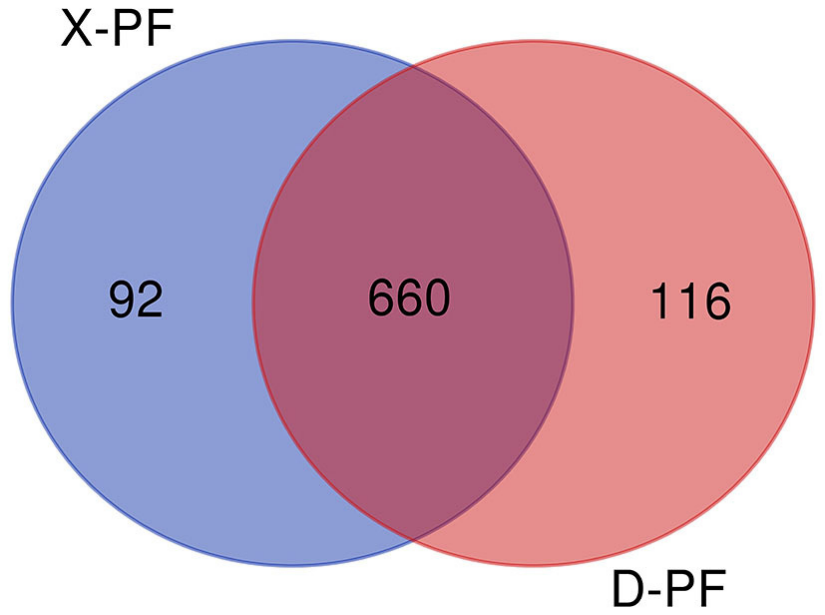
miRNAs Venn diagram between two groups (D-PF vs. X-PF). D-PF represented the subcutaneous adipose tissue of Duolang sheep, X-PF represented the subcutaneous adipose tissue of Small Tail Han sheep.

After comparing with miRBase database, we obtain the known miRNA information of each sample, and then compare and analyze the sequences without annotation information to obtain the newly predicted miRNA. A total of 868 miRNAs were obtained in this experiment, of which the number of known and new miRNAs were 149 and 719, respectively, 660 of the 868 miRNAs were coexpressed, and 116 and 92 miRNAs existed only in Duolang sheep and Small Tail Han sheep, respectively (Figure 1). The statistics of small RNA are shown in Table 3, including miRNA sequences and non-miRNA sequences. miRNA accounts for the largest proportion in each sample (Supplementary Figure S1).

**Table 3:** Small RNA classification statistics.

**Type**	**D_PF_1**	**D_PF_2**	**D_PF_3**	**X_PF_1**	**X_PF_2**	**X_PF_3**
Known miRNA	13,353,021 (71.34%)	14,835,693 (77.92%)	13,060,553 (73.52%)	13,224,849 (71.32%)	16,825,434 (74.94%)	12,843,282 (67.79%)
Novel miRNA	798,022 (4.26%)	892,391 (4.69%)	735,426 (4.14%)	1,027,702 (5.54%)	1,330,213 (5.93%)	881,219 (4.65%)
rRNA	305,343 (1.63%)	91,711 (0.48%)	179,636 (1.01%)	149,916 (0.81%)	119,537 (0.53%)	184,698 (0.97%)
tRNA	532,977 (2.85%)	189,584 (1.0%)	405,022 (2.28%)	255,955 (1.38%)	951,902 (4.24%)	645,567 (3.41%)
snoRNA	3,185 (0.02%)	2,976 (0.02%)	4,895 (0.03%)	3,763 (0.02%)	3,455 (0.02%)	2,887 (0.02%)
snRNA	4,902 (0.03%)	4,423 (0.02%)	3,732 (0.02%)	2,679 (0.01%)	2,968 (0.01%)	4,771 (0.03%)
Unknown	863,087 (4.61%)	508,884 (2.67%)	697,672 (3.93%)	583,771 (3.15%)	504,830 (2.25%)	764,738 (4.04%)
Other	2,856,372 (15.27%)	2,514,999 (13.21%)	2,677,669 (15.07%)	3,293,946 (17.77%)	2,712,141 (12.08%)	3,617,872 (19.1%)
Sum	18,716,909	19,040,661	17,764,605	18,542,581	22,450,480	18,945,034

### miRNA Structural Analysis

**Figure 2:**
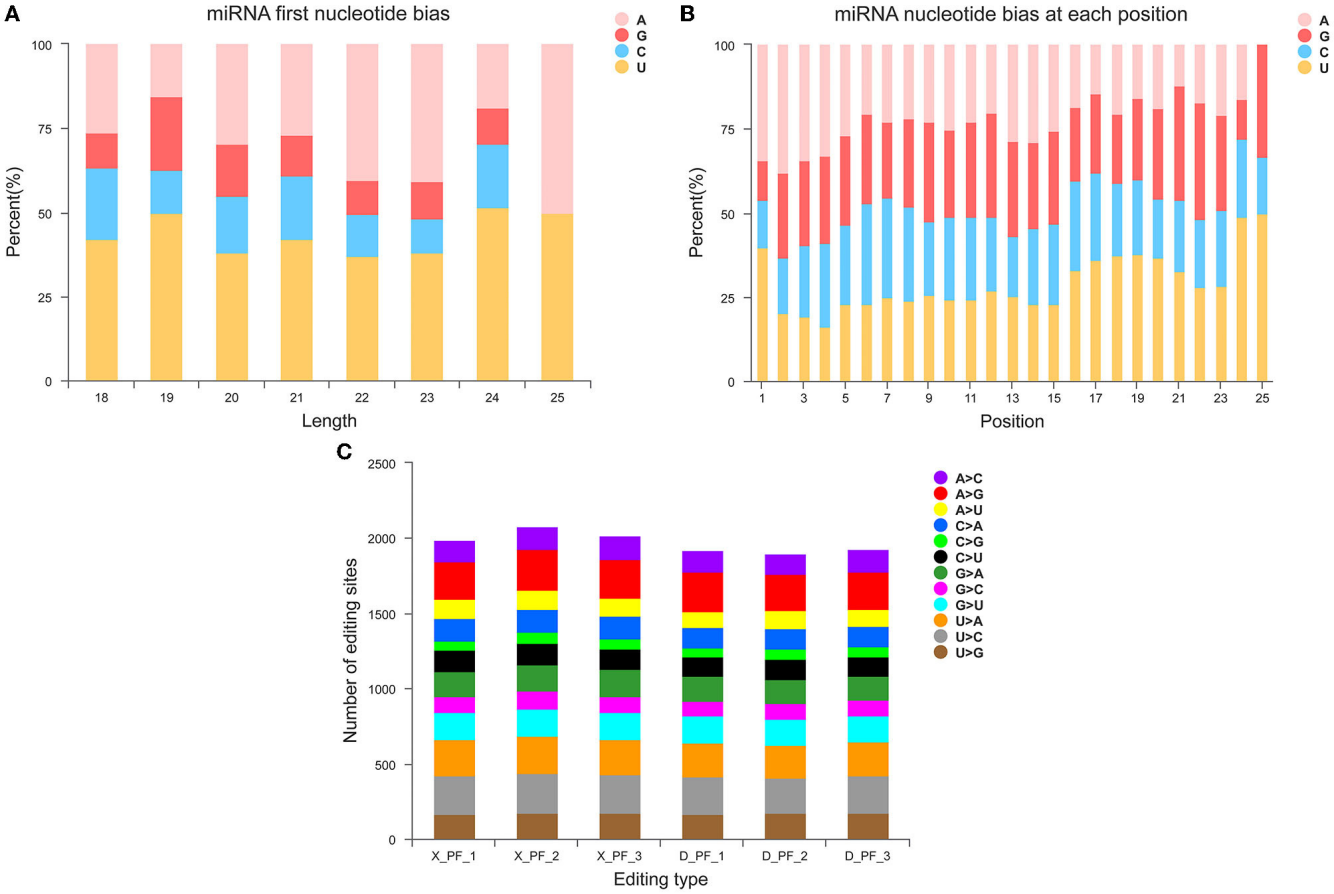
Structural characteristics of miRNA. **(A)** First base preference distribution map of miRNAs with different lengths. **(B)** Distribution map of base preference at different miRNA sites. **(C)** miRNA base editing type distribution map.

Counting the known 149 miRNAs, we found them from 51 families and 271 species (Supplementary Table S2). Statistics on the first base of miRNAs of different lengths and base preferences at different sites (Figure 2A) and base preferences at different miRNA sites (Figure 2B), we found that four bases A, G, C, and U accounted for the proportion of first place varies, and the proportion of first place preference U is the largest (Supplementary Table S3), which is consistent with previous studies in pigs. Analysis of miRNA base editing in each sample showed that the number of various base editing sites in the subcutaneous adipose tissue of the Small Tail Han sheep was slightly higher than that of the Duolang sheep (Figure 2C).

### Differential Expression of miRNA

**Figure 3:**
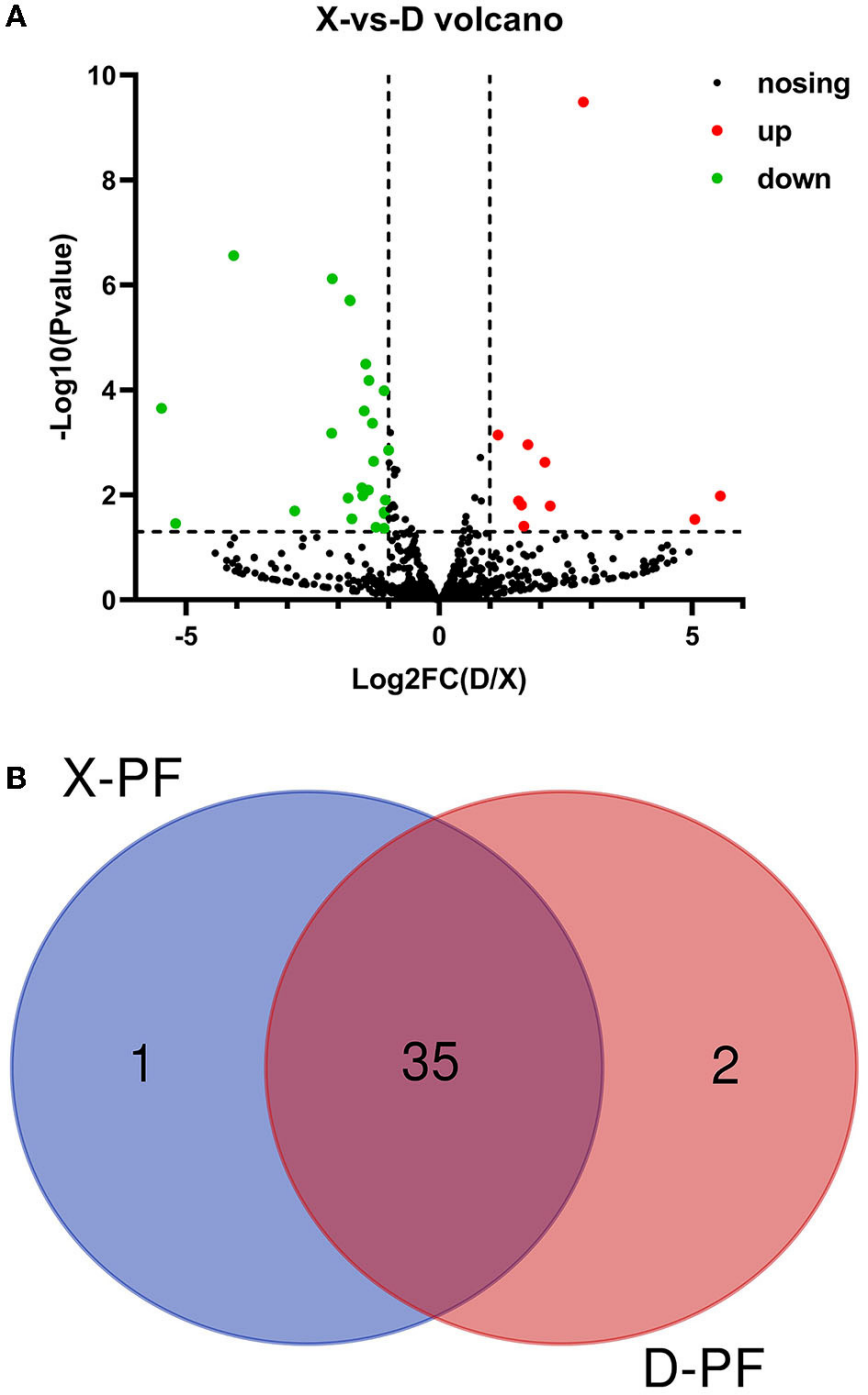
Differential expression of miRNA. **(A)** Volcano map of differentially expressed miRNAs. The scatter in the figure represents the miRNAs, the black dots indicate the miRNAs with no significant differences, the red dots indicating a significant up-regulation of the miRNA, and the green dots represent a significant down-regulation of the miRNA. **(B)** The miRNA Venn diagram was differentially expressed in the two groups. D-PF represented the subcutaneous adipose tissue of Duolang sheep, X-PF represented the subcutaneous adipose tissue of Small Tail Han sheep.

There are obvious differences in the deposition of adipose tissue between Duolang sheep and Small Tail Han sheep. By comparing the miRNAs expression of the two breeds of sheep, the differentially expressed miRNAs in the subcutaneous fat of the two breeds of sheep were screened. About 95% of the miRNAs were filtered out. There were 38 remaining differentially expressed miRNAs, of which 9 were known miRNAs and 29 were unknown miRNAs. Among the differentially expressed miRNAs, 10 were up-regulated and 28 were down-regulated in Duolang sheep (Figure 3A; Supplementary Table S4). To further analyze the interaction of the differentially expressed miRNAs in the two breeds, we constructed a Venn diagram (Figure 3B), of which one was only present in the Small Tail Han sheep and two were only in the Duolang sheep.

### miRNA Target Gene Prediction and Functional Enrichment Analysis

**Figure 4:**
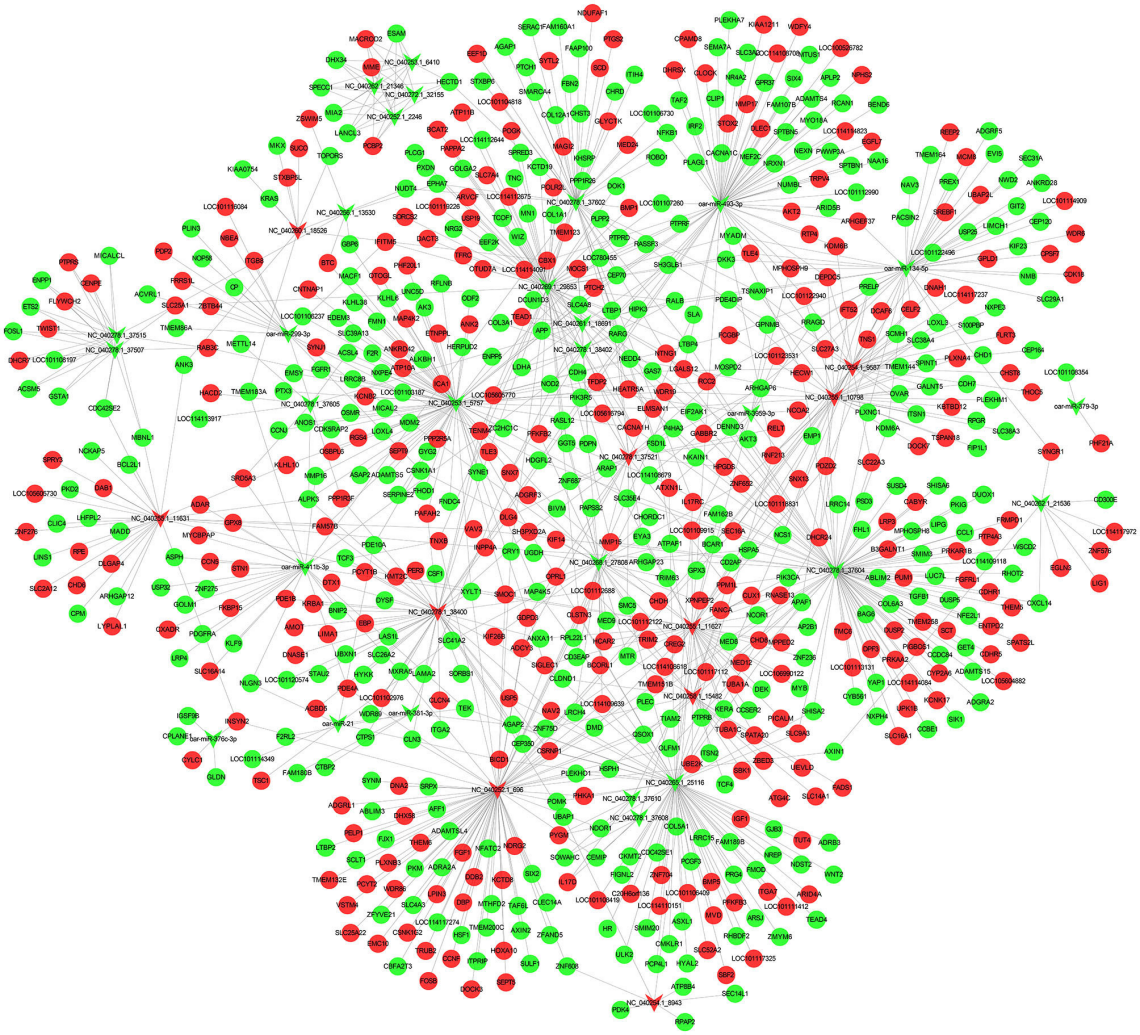
Regulatory networks of genes. miRNA-mRNA regulatory network in sheep subcutaneous adipose tissue. The circle represents mRNA, and the arrow represents miRNA, the red represents up-regulated expression in Duolang sheep, and the green represents down-regulated expression.

miRNA functions by inhibiting the translation of target genes. In order to more directly observe the interaction between miRNAs and mRNAs, we useed Cytoscape to draw. When predicting the target genes of 38 differentially expressed miRNAs, we found that there are a large number of target genes, a total of 854 (Figure 4; Supplementary Table S5). The results show that one miRNA may regulate multiple target genes, and a target gene may also be regulated by multiple different kinds of miRNAs.

**Figure 5:**
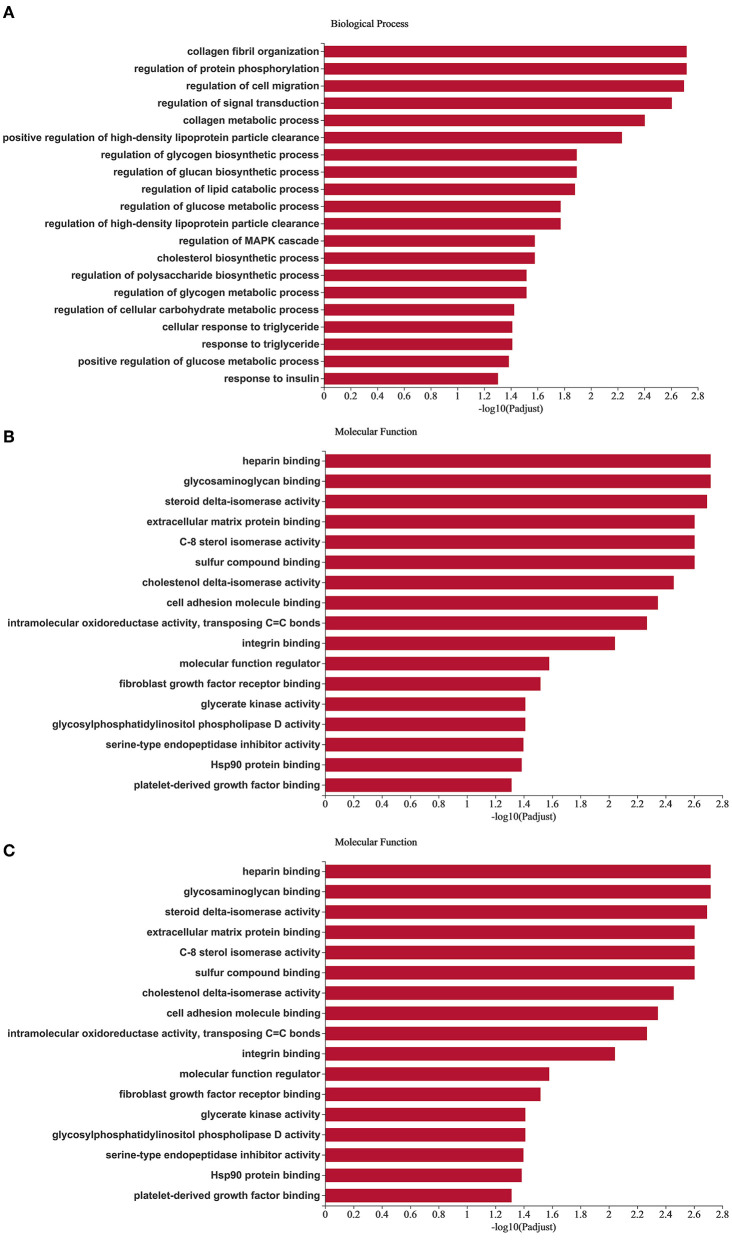
GO enrichment analysis of target genes. **(A–C)** Are biological processes, molecular function, and cellular component, respectively. The X-axis is the enrichment significance, and the Y-axis is GO term.

In order to explore the function of miRNAs, we followed the GO enrichment analysis of the target mRNAs of 38 different miRNAs. The results showed that in the biological process (Figure 5A), the target genes were significantly enriched in the regulation of protein phosphorylation, regulation of lipid catabolic process, regulation of MAPK cascade, cholesterol biosynthetic process, response to insulin. Differentially expressed target genes in molecular function (Figure 5B), mainly enriched in heparin binding, glycosaminoglycan binding, C-8 sterol isomerase activity, glycerate kinase activity and glycosylphosphatidylinositol phospholipase D activity. In terms of cellular components (Figure 5C), it was significantly enriched in cell surface, extracellular matrix, receptor complex and other related items. GO analysis showed that the differential miRNAs mainly regulated the lipid metabolism process and the regulation of enzyme activities in Duolang sheep and Small Tail Han sheep, resulting in differences in fat deposition between the two breeds.

**Figure 6:**
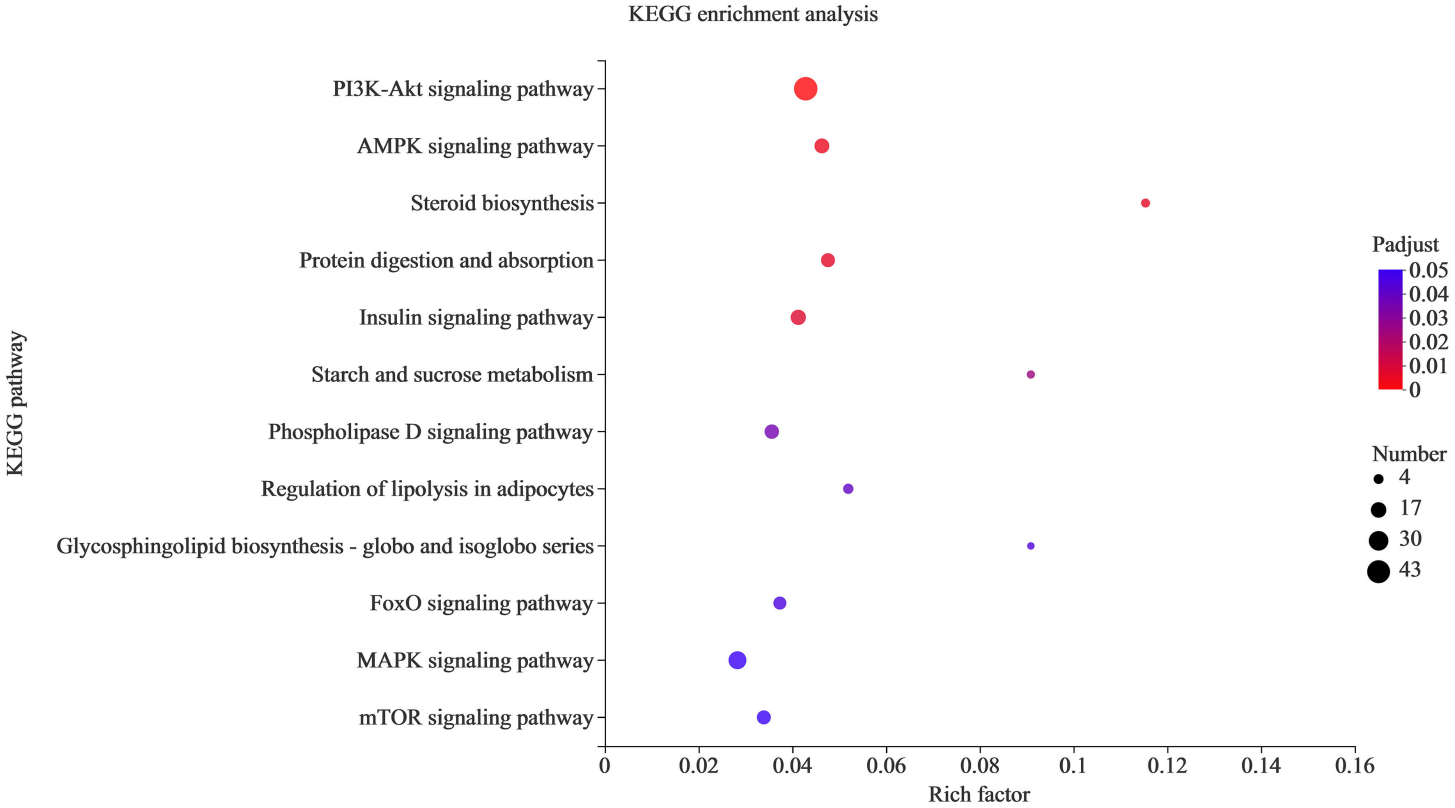
KEGG pathway analysis of the target genes. The X-axis is the rich factor, and the Y-axis is KEGG pathway.

KEGG enrichment was used to analyze the function of target genes (Figure 6). A total of 284 pathways were enriched, and the corrected *p*-adjust < 0.05 was set to screen the pathways. Among them, the significantly enriched pathways were PI3K Akt signaling pathway, AMPK, steroid biosynthesis, insulin signaling pathway, regulation of lipolysis in adipocytes, phospholipase D signaling pathway, MAPK and other pathways related to lipid metabolism. Therefore, it is speculated that the differential expression of miRNAs may regulate fat deposition by changing the signal transduction of two breeds of sheep.

### The Relationship Between miRNA and Target Genes in Regulation of Lipolysis in Adipocytes

**Figure 7:**
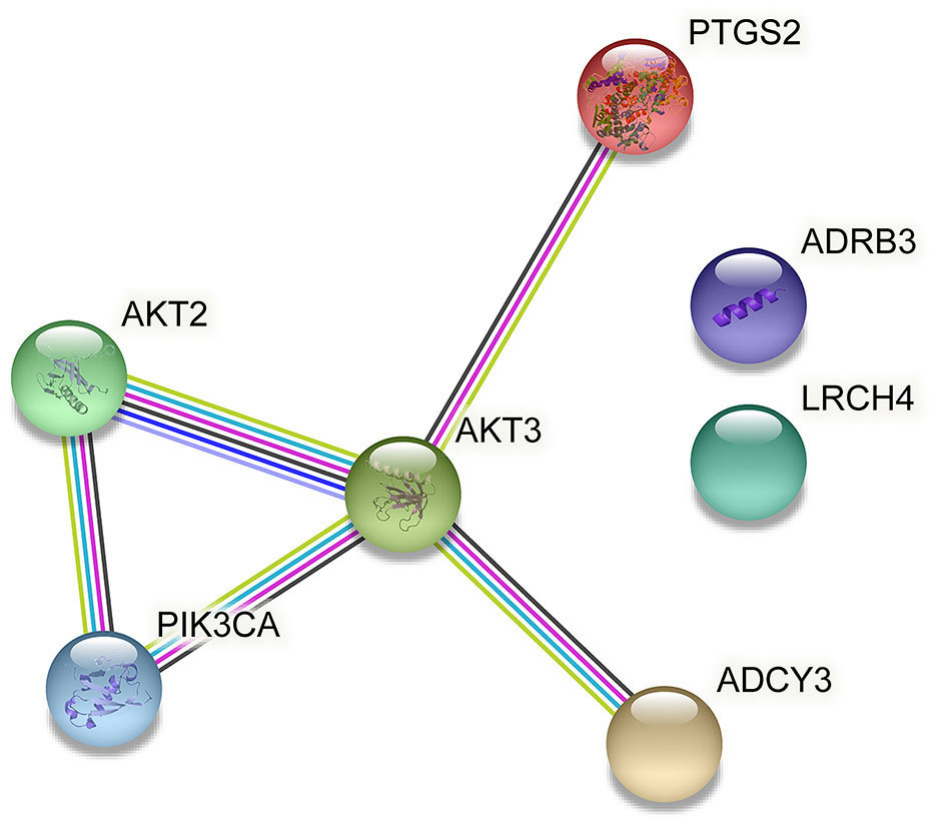
Gene interactions enriched in Regulation of lipolysis in adipocytes.

**Figure 8:**
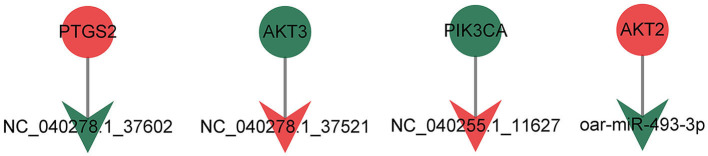
The targeting relationship of genes in Regulation of lipolysis in adipocytes.

Regulation of lipolysis in adipocytes plays an important role in the process of fat deposition. We found that miRNA target genes are partially enriched in this signaling pathway, including PTGS2, ADRB3, LRCH4, PIK3CA, ADCY3, AKT3 and AKT2. The query results based on string database show that there is an interaction relationship between PTGS2, AKT2, AKT3 and PIK3CA (Figure 7). The miRNAs that target and regulate them are NC_040278.1_37602, oar-miR-493-3p, NC_040255.1_11627 and NC_040278.1_37521 (Figure 8). These differentially expressed miRNAs may cause the difference in fat deposition between Duolang sheep and Small Tail Han sheep.

### QRT-PCR

**Figure 9:**
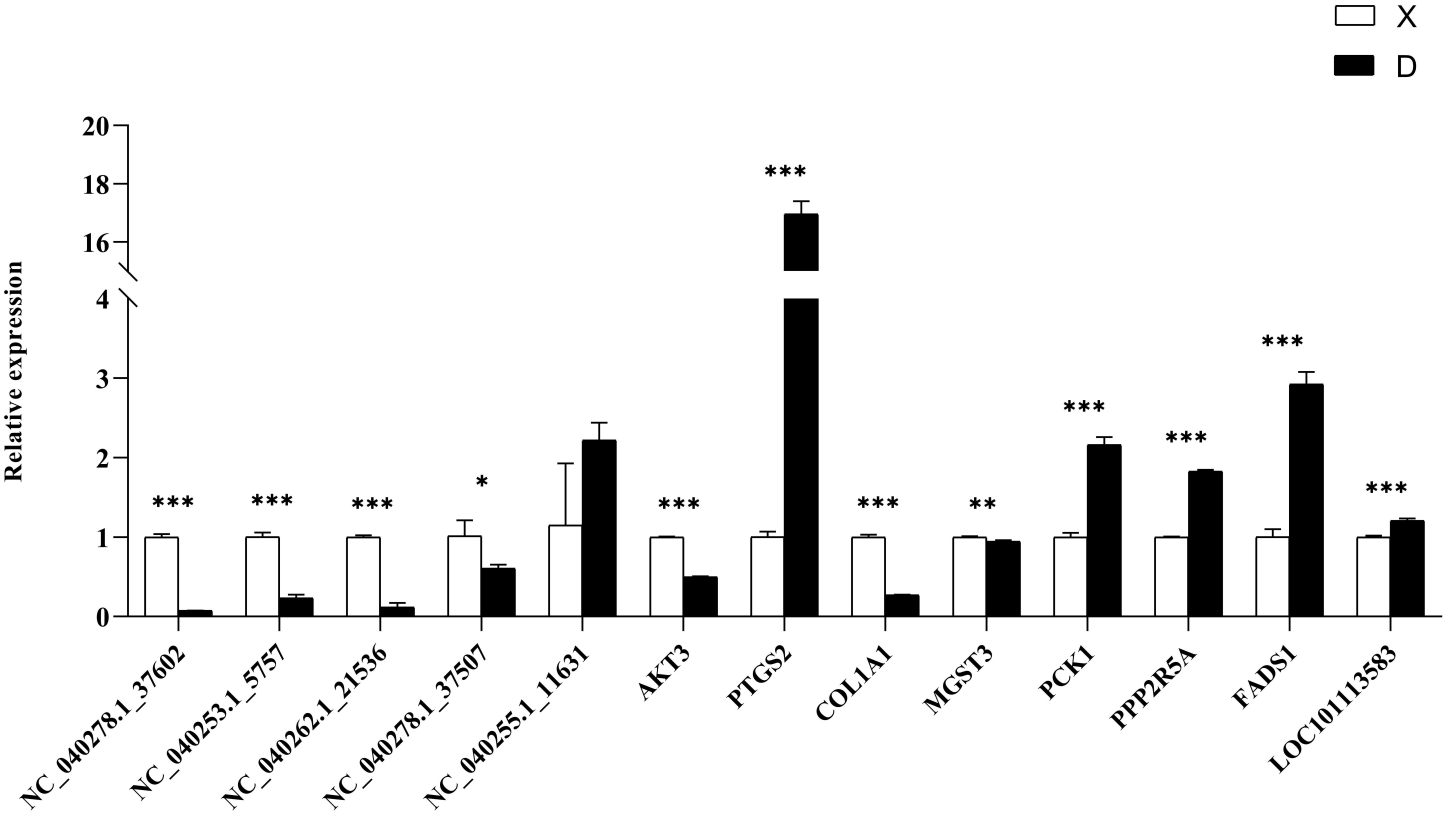
qRT-PCR verification of differentially expressed genes. The differential expression of genes in subcutaneous adipose tissues between Duolang and Small Tail Han sheep was verified qRT-PCR. *P < 0.05; **P < 0.01; ***P < 0.001.

To verify the accuracy of the RNA-Seq data, we detected the relative expression levels of 13 transcripts by qRT-PCR (Figure 9). The results showed that four miRNAs (NC_040278.1_37602, NC_040253.1_5757, NC_040262.1_21536 and NC_040278.1_37507) were down-regulated and NC_040255.1_11631 was up-regulated, which was consistent with the RNA-Seq results. In addition, we verified the expression of target genes (AKT3, PTGS2, COL1A1, MGST3, PCK1, PPP2R5A, FADS1, and LOC101113583) and proved that the sequencing results were reliable.

## Discussion

In recent years, related research on ncRNA in cancer has been widely reported. miRNA is a short-chain non-coding RNA that can specifically bind to the 3′ UTR of mRNA to inhibit mRNA translation or promote mRNA degradation. The deposition of adipose tissue not only plays a role in storing energy, but also can improve slaughter weight, slaughter rate, carcass quality and economic benefits for livestock and poultry in the fattening period. Previous studies on miRNA have mostly appeared in model animals such as mice, or in pigs that are genetically similar to humans, and there is relatively little research on miRNA in sheep. In this study, Duolang sheep and Small Tail Han sheep with different fat deposition ability were used to explore the regulation mechanism of miRNAs on fat deposition. We sequenced the miRNAs in sheep subcutaneous adipose tissue and analyzed the obtained data. The results showed that the structural characteristics of miRNAs were consistent with previous studies in other animals. And a total of 38 differentially expressed miRNAs were obtained, including 29 new miRNAs. MiRanda software was used to predict the target genes of miRNAs. The target genes were analyzed by GO and KEGG function enrichment, so as to speculate the function of miRNA. The target genes were mainly involved in regulation of lipolysis in adipocytes. The above results showed that miRNAs played a regulatory role in the deposition of sheep adipose tissue. Finally, the sequencing reliability of 13 RNAs were further verified by qRT-PCR reaction. In sum, this study explored the role of miRNAs in regulating subcutaneous fat deposition in sheep and might provide a basis for studying the molecular mechanism of miRNAs regulating lipid metabolism.

Alterations in lipolysis are often associated with obesity, including increased rates of basal lipolysis, which may contribute to the development of insulin resistance, and impaired responses to stimulate lipolysis. Obesity is characterized by excess white adipose tissue and enlarged adipocyte size due to increased triacylglycerol storage. This study found that PTGS2, AKT2, AKT3, and PIK3CA were significantly enriched in the regulation of lipolysis in adipocytes. These 4 differentially expressed genes were regulated by 4 miRNAs (NC_ 040278.1_ 37602, oar-mir-493-3p, NC_ 040278.1_ 37521 and NC_ 040255.1_ 11627). The unknown miRNA NC_040278.1_37602 was down-regulated in subcutaneous adipose tissue of Duolang sheep. It might target PTGS2 and involve in regulation of lipolysis in adipocytes pathway, thus regulating fat deposition. Fu et al. (44) found that the differentially expressed gene PTGS2 was significantly enriched in the arachidonic acid metabolism signaling pathway in high-fat diet-induced obesity mice, and these genes were closely related to glycolipid metabolism in adipose tissue. Fluoxetine has been shown to increase *in vivo* and *in vitro* hepatic lipid accumulation. Fluoxetine treatment increased mRNA expression of prostaglandin biosynthetic enzymes PTGS2 (45). The differentially expressed mRNA was combined with miRNA using the mode of up-down or down-up to construct the regulatory network. In this study, PTGS2 was up-regulated in subcutaneous adipose tissue of Duolang sheep, and it was speculated that NC_040278.1_37602 could affect the deposition of sheep subcutaneous adipose tissue by regulating PTGS2.

The oar-mir-493-3p was down-regulated in subcutaneous adipose tissue of Duolang sheep. It has been found that ssc-mir-493 is highly expressed in the skeletal muscle of Duroc pigs and interacts with PDK4 (46). PDK4 (47) is located in the mitochondrial matrix and inhibits the pyruvate dehydrogenase complex, thereby catalyzing the conversion of pyruvate to acetylcoa. Therefore, it is responsible for reducing the utilization of glucose and the up regulation of fatty acid oxidation. It is speculated that mir-493 may be involved in the regulation of lipid metabolism by targeting PDK4. Some studies have analyzed the miRNAs in human islets, liver and skeletal muscle, and found that mir-493 is also enriched in islets, and the expression level is higher than that in liver and skeletal muscle (48). Other studies have found that mir-493 can regulate IGF1R and its downstream effector molecule mapk1. These may lay a foundation for revealing the potential regulatory mechanism of mir-493 in affecting lipid metabolism and related diseases by affecting insulin synthesis and secretion. In this study, oar-mir-493-3p might target AKT2 and involve in regulation of lipolysis in adipocytes pathway, thus regulating fat deposition. AKT, also known as protein kinase B, includes three closely related isoforms, AKT1, AKT2 and AKT3. AKT2 is abundant in brown fat and is up-regulated in 3T3-L1 adipocytes (49) and is critical for adipose tissue growth, not because it controls differentiation per se, but because it promotes lipid accumulation (50). The study found that BCAAs are associated with obesity-related metabolic disorders, BCAAs aggravate obesity-related hepatic glucolipid metabolism disorders by weakening AKT2 signaling pathway and supplementing BCAAs significantly increased hepatic gluconeogenesis in high-fat diet-induced obese mice and inhibited hepatic lipogenesis. This resulted from severe attenuation of AKT2 signaling through mTORC1 and mTORC2-dependent pathways. BCKAs suppressed AKT2 activation through mTORC1 and mTORC2 signaling and promote AKT2 ubiquitin-proteasome-dependent degradation through the mTORC2 pathway (51). In this experiment, AKT2 was up-regulated in the subcutaneous adipose tissue of Duolang sheep, and oar-mir-493-3p regulated the subcutaneous adipose tissue deposition of sheep by targeting AKT2.

The unknown miRNA NC_040278.1_37521 was up-regulated in subcutaneous adipose tissue of Duolang sheep, and it might target AKT3 and involve in regulation of lipolysis in adipocytes pathway. Studies have found that AKT3 specifically phosphorylates WNK1 at T58. Lack of AKT3 in adipocytes increases WNK1 protein levels, leading to the activation of SGK1 (52). In turn, SGK1 promotes adipogenesis by phosphorylating and inhibiting the transcription factor FOXO1, which in turn activates the transcription of PPARγ in adipocytes, thereby promoting adipogenesis, and mice lacking AKT3 have increased adipocyte numbers, white adipose tissue expansion when fed a high-fat diet, and glucose homeostasis is impaired. Thus, the interaction between AKT3, WNK1 and SGK1 regulates adipogenesis *in vivo*, and dysregulation of this pathway can lead to increased adipogenesis and obesity as well as insulin resistance (53). AKT3 was down-regulated in subcutaneous adipose tissue of Duolang sheep, and NC_040278.1_37521 affects the deposition of adipose tissue by regulating it.

The unknown miRNA NC_040255.1_11627 was up-regulated in subcutaneous adipose tissue of Duolang sheep, and it might target PIK3CA and involve in regulation of lipolysis in adipocytes pathway. The protein encoded by the PIK3CA gene is the catalytic subunit of PI3Ks, a family of lipid kinases that can specifically phosphorylate the 3-hydroxyl group of phosphatidylinositol to produce second messenger inositols (54). NC_040255.1_11627 functions by regulating PIK3CA. PIK3CA was also significantly enriched in signaling pathways such as PI3K-Akt and mTOR in this study. More and more studies have shown that the mTOR signaling pathway plays an important role in maintaining energy homeostasis and glucose and lipid metabolism. Its activation can lead to obesity. As a downstream target of the PI3K/Akt pathway, mTOR can regulate lipid synthesis through PPARγ and SREBP1 (55). Activation of AMPK inhibits the activity of mTOCR1 (56). AMPK can phosphorylate mTOCR1 to promote glucose catabolism, fatty acid oxidation and autophagy, and inhibit fatty acid and protein synthesis (57). We therefore selected miRNAs (NC_040278.1_37602, oar-miR-493-3p, NC_040278.1_37521 and NC_040255.1_11627) and target genes (PTGS2, AKT2, AKT3, and PIK3CA) as important candidate genes by functional analysis. The analysis identified pathways related to fat metabolism, including regulation of lipolysis in adipocytes, that may indirectly affect subcutaneous adipose tissue deposition in sheep.

## Conclusions

In summary, miRNAs could regulate fat deposition in Duolang and Small Tail Han sheep. According to the results of RNA-Seq, miRNAs can target mRNAs and then play an important role in regulation of lipolysis in adipocytes. By this way, NC_040278.1_37602, oar-miR-493-3p, NC_040278.1_37521, and NC_040255.1_11627 might target PTGS2, AKT2, AKT3, and PIK3CA, respectively, play key regulatory roles. This study can provides valuable information to supplement the sheep miRNA database and further study the biology of sheep subcutaneous fat miRNA. Moreover, it lays a foundation for exploring the mechanism of fat deposition in sheep, which was beneficial to the development of animal husbandry.

## Data Availability Statement

The RNA-Seq data have been deposited in the NCBI Sequence Read Archive (SRA) database with accession number PRJNA801884.

## Ethics Statement

All the procedures involving animals were approved by the animal care and use committee at the Institute of Animal Sciences, Chinese Academy of Agricultural Sciences (NO. IAS2019-82), where the study was conducted. All the experiments were performed in accordance with the relevant guidelines and regulations set by the Ministry of Agriculture of the People's Republic of China.

## Author Contributions

T-YL, HF, SY, and L-LX performed the experiment. T-YL analyzed the data. HF, SY, and L-LX interpreted the data. X-YM conceived and designed the study. T-YL and X-YM wrote the paper. All authors read and approved the final manuscript.

## Funding

This work was supported by a grant from the Major Science and Technology Project of New Variety Breeding of Genetically Modified Organisms (Nos. 2009ZX08008-004 and 2008ZX08008-003), the Agricultural Science and Technology Innovation Programme (ASTIPIAS05) and the Basic Research Fund for Central Public Research Institutes of CAAS (Y2016JC22, Y2018PT68).

## Conflict of Interest

The authors declare that the research was conducted in the absence of any commercial or financial relationships that could be construed as a potential conflict of interest.

## Publisher's Note

All claims expressed in this article are solely those of the authors and do not necessarily represent those of their affiliated organizations, or those of the publisher, the editors and the reviewers. Any product that may be evaluated in this article, or claim that may be made by its manufacturer, is not guaranteed or endorsed by the publisher.
