# Supplementary material for: Genome-Wide Analysis of microRNAs Identifies the Lipid Metabolism Pathway to Be a Defining Factor in Adipose Tissue From Different Sheep
Source: Front Vet Sci. 2022 Jul 8;9:938311. doi: 10.3389/fvets.2022.938311 (PMC9308008; doi:10.3389/fvets.2022.938311)
Supplement: Supplementary file 1 [file Data_Sheet_1.zip › 938311_SupMaterial/Data Sheet 1/Supplementary table and figure/Supplementary figure/Supplementary Figure.docx]

**Supplementary Figure**

**
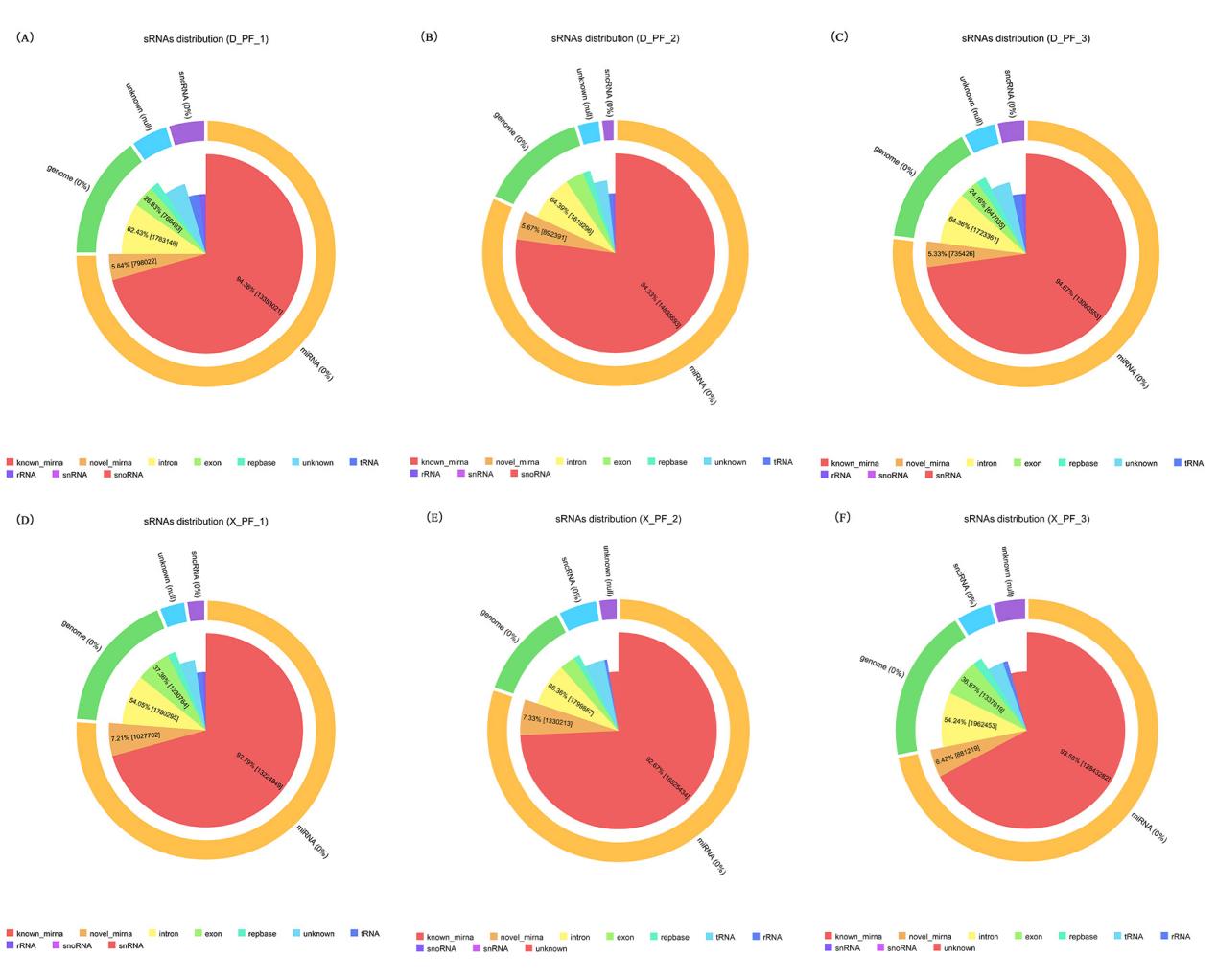
**

Supplementary Figure S1 | SRNA analysis in adipose tissue of Duolang sheep and Small Tail Han sheep.
