# Supplementary figures and images for: Genome-Wide Analysis of microRNAs Identifies the Lipid Metabolism Pathway to Be a Defining Factor in Adipose Tissue From Different Sheep
Source: Front Vet Sci. 2022 Jul 8;9:938311. doi: 10.3389/fvets.2022.938311 (PMC9308008; doi:10.3389/fvets.2022.938311)

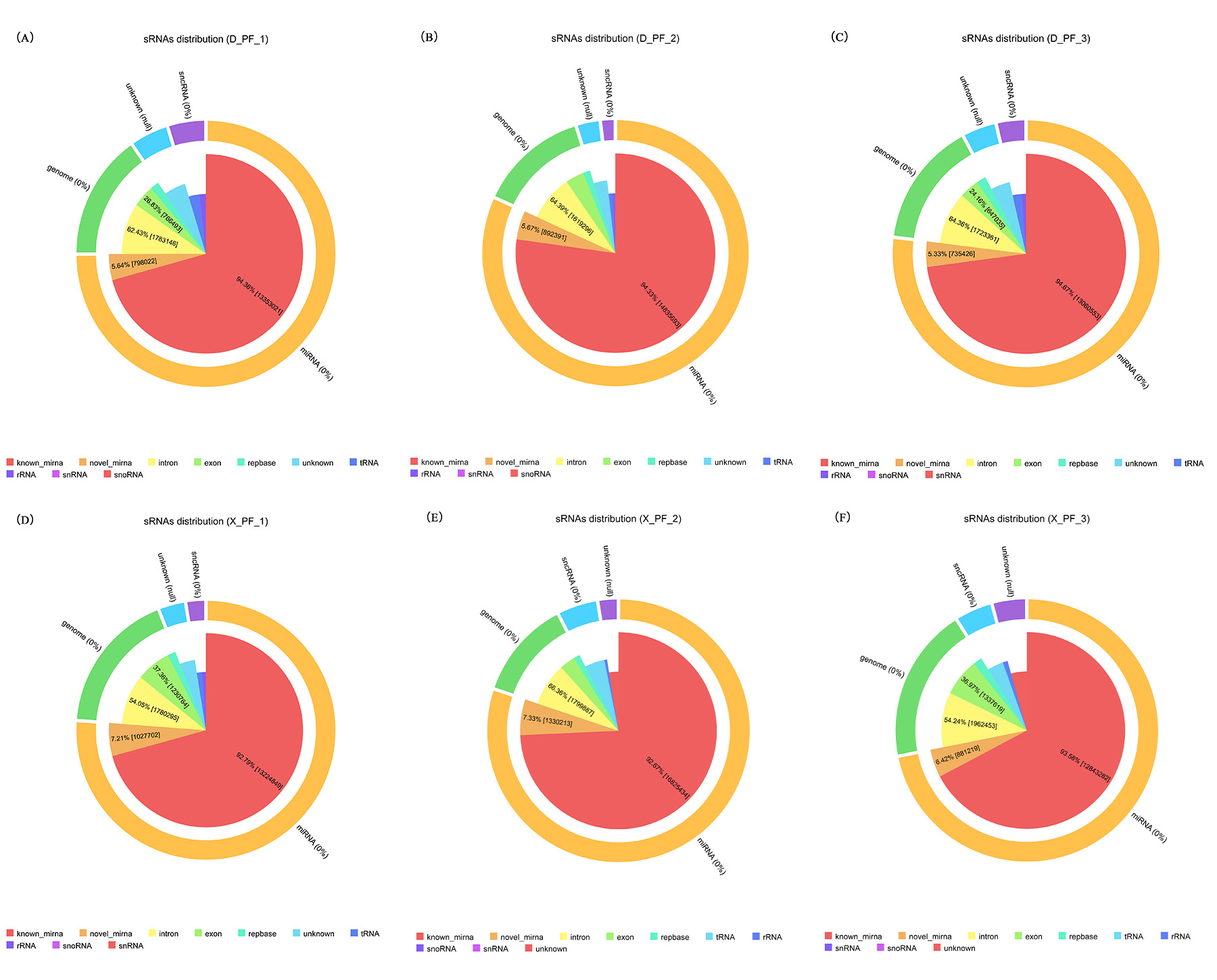

Supplement: Supplementary file 1 [file Data_Sheet_1.zip › 938311_SupMaterial/Data Sheet 1/Supplementary table and figure/Supplementary figure/Supplementary Figure 1.jpg]

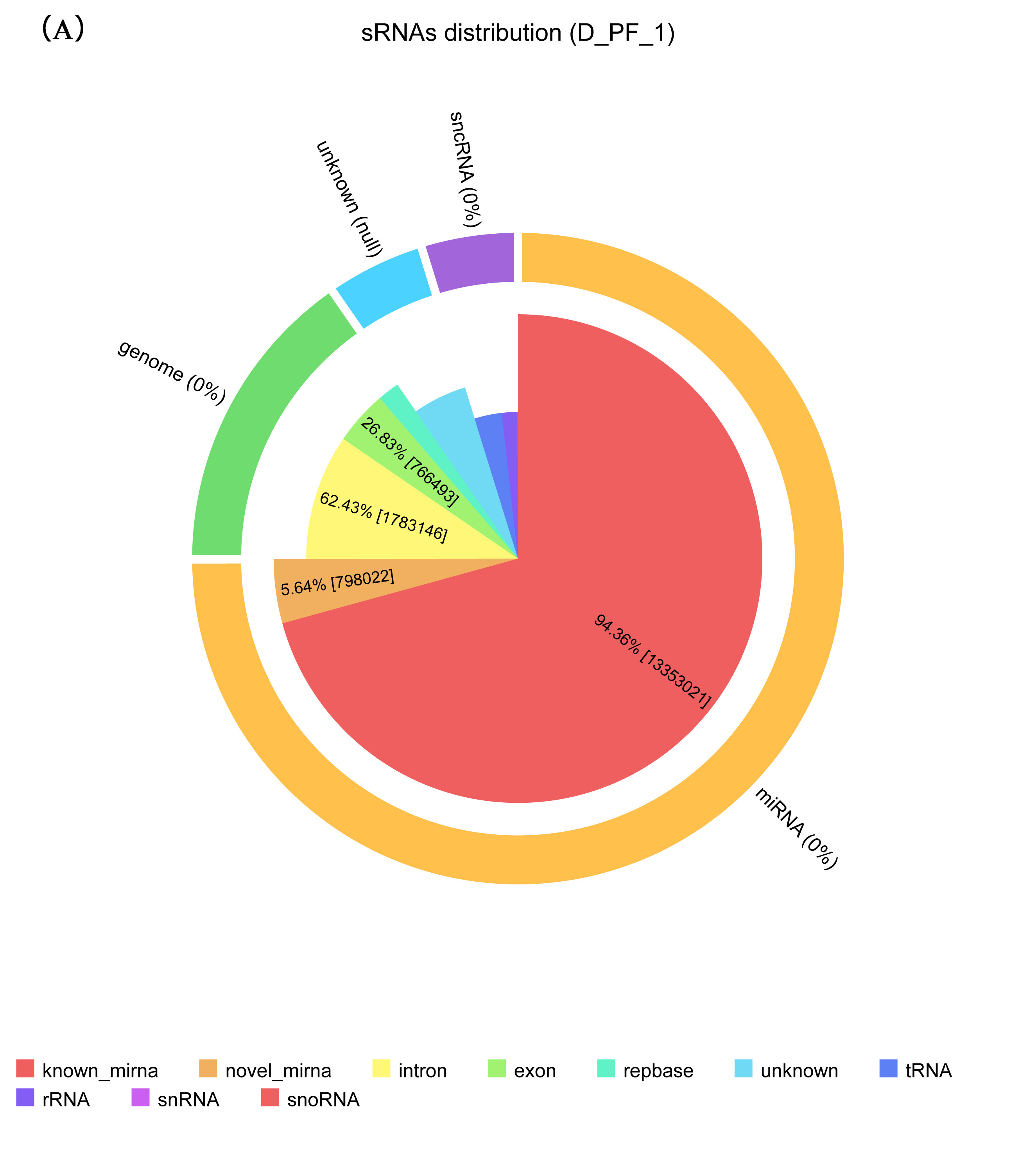

Supplement: Supplementary file 1 [file Data_Sheet_1.zip › 938311_SupMaterial/Data Sheet 1/Supplementary table and figure/Supplementary figure/sRNAs-distribution-(D-PF-1).jpg]

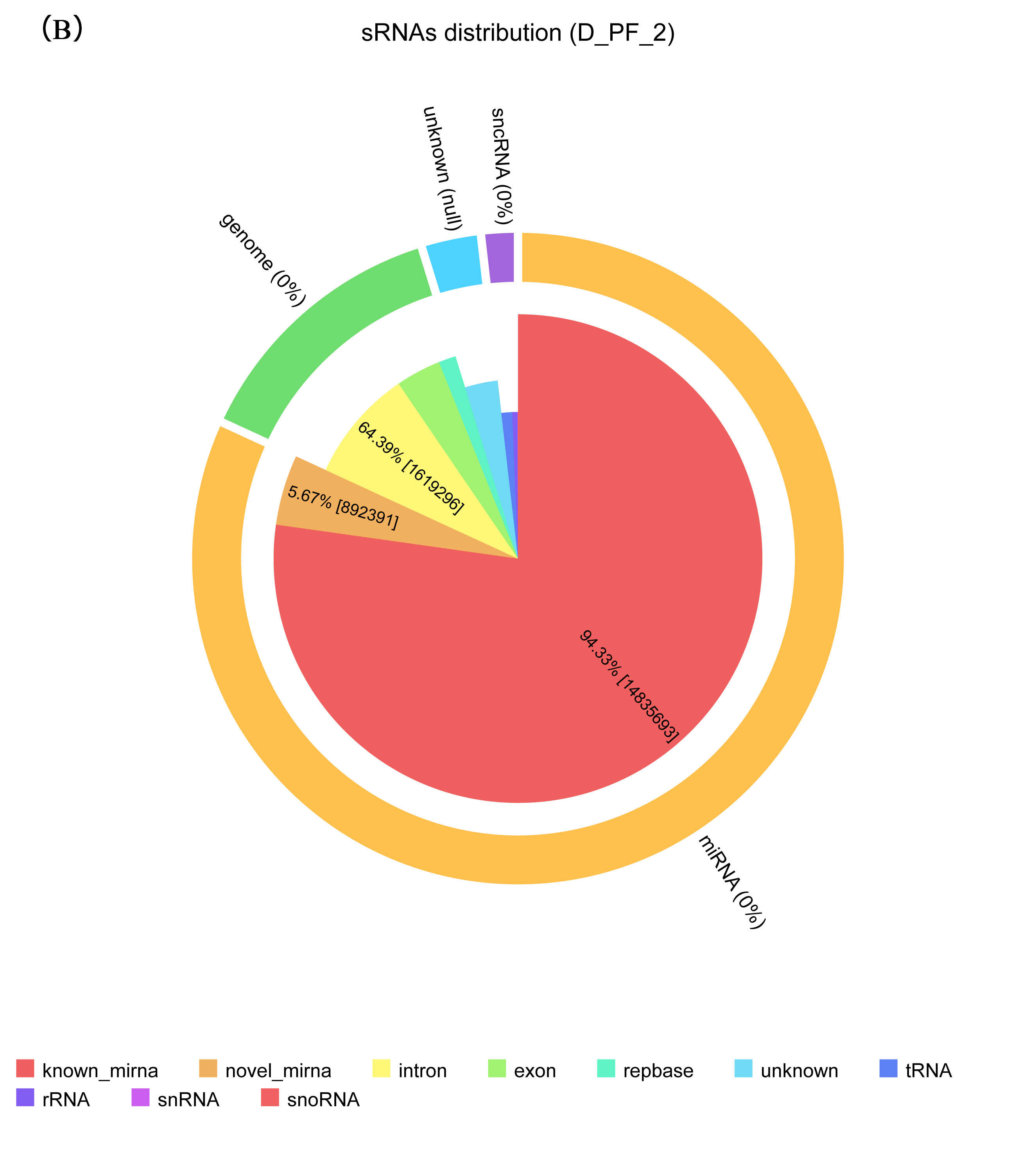

Supplement: Supplementary file 1 [file Data_Sheet_1.zip › 938311_SupMaterial/Data Sheet 1/Supplementary table and figure/Supplementary figure/sRNAs-distribution-(D-PF-2).jpg]

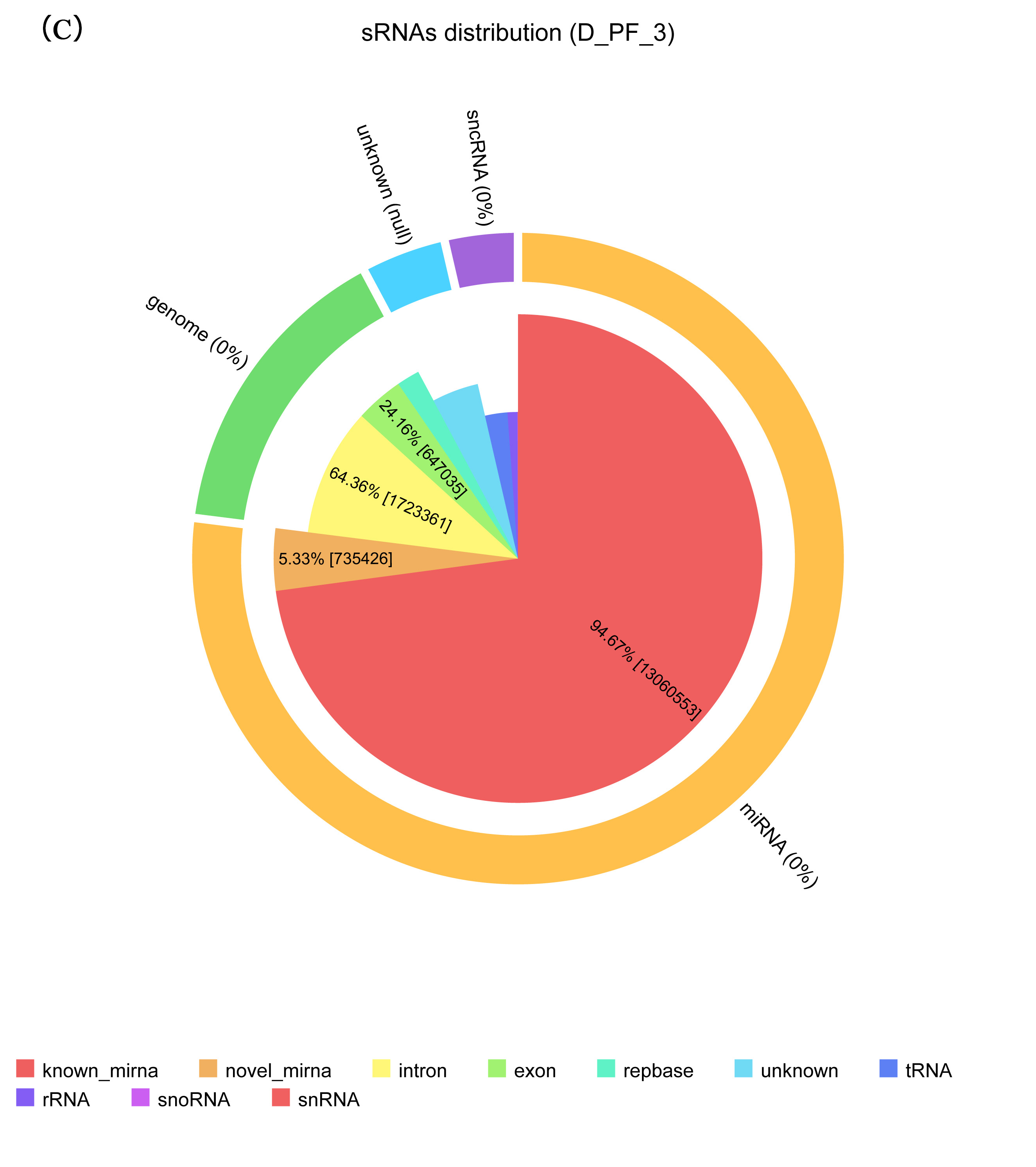

Supplement: Supplementary file 1 [file Data_Sheet_1.zip › 938311_SupMaterial/Data Sheet 1/Supplementary table and figure/Supplementary figure/sRNAs-distribution-(D-PF-3).jpg]

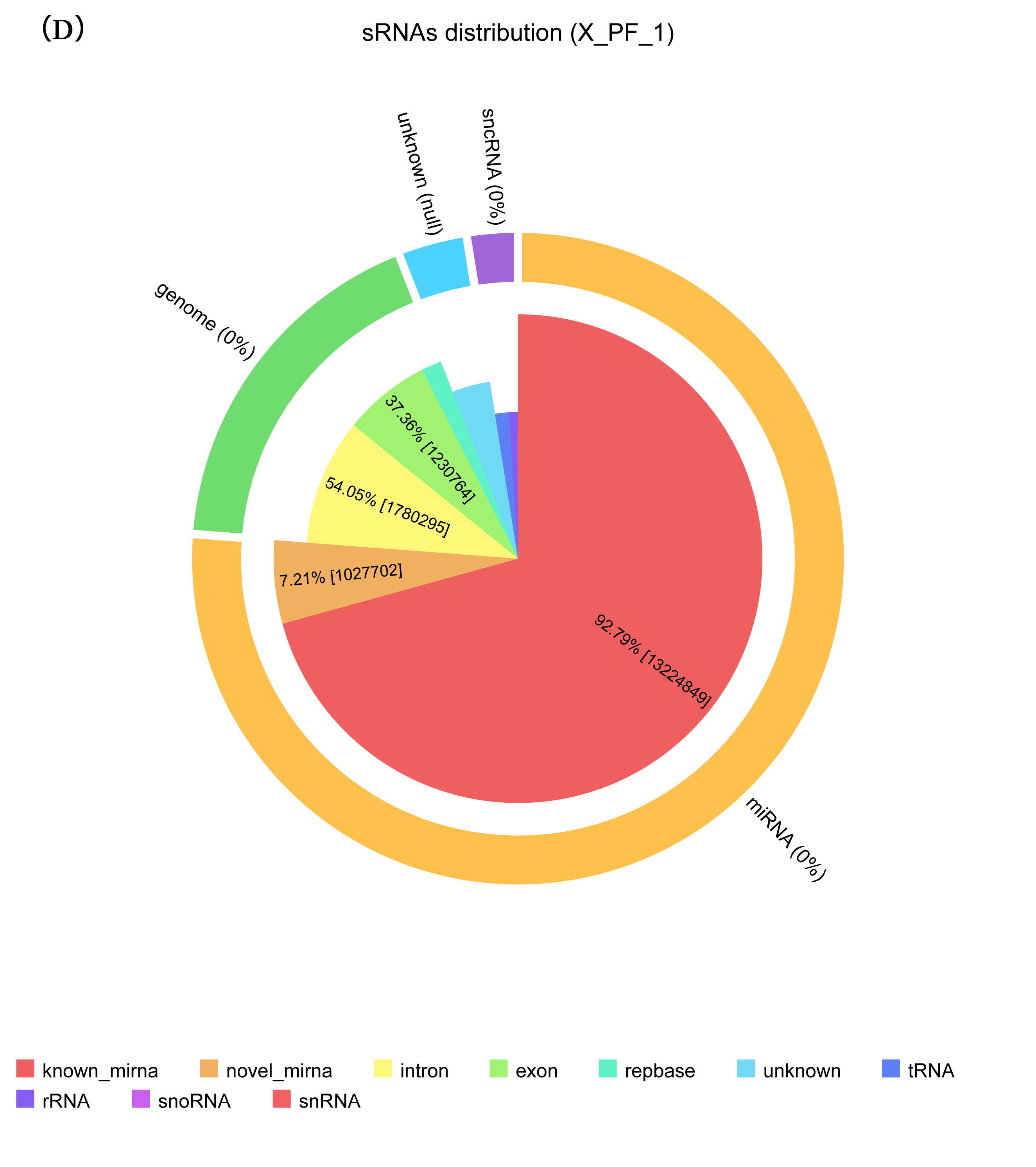

Supplement: Supplementary file 1 [file Data_Sheet_1.zip › 938311_SupMaterial/Data Sheet 1/Supplementary table and figure/Supplementary figure/sRNAs-distribution-(X-PF-1).jpg]

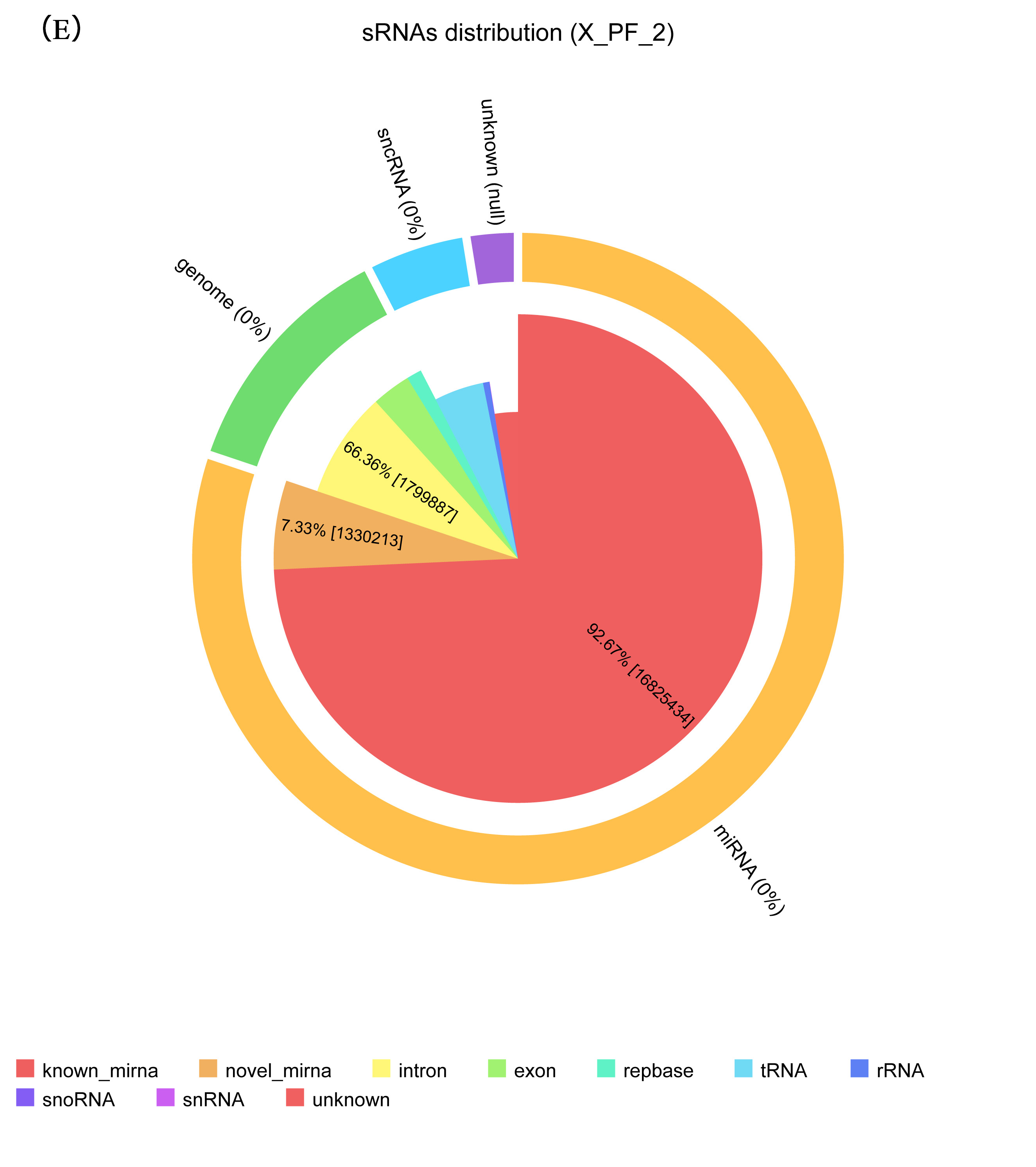

Supplement: Supplementary file 1 [file Data_Sheet_1.zip › 938311_SupMaterial/Data Sheet 1/Supplementary table and figure/Supplementary figure/sRNAs-distribution-(X-PF-2).jpg]

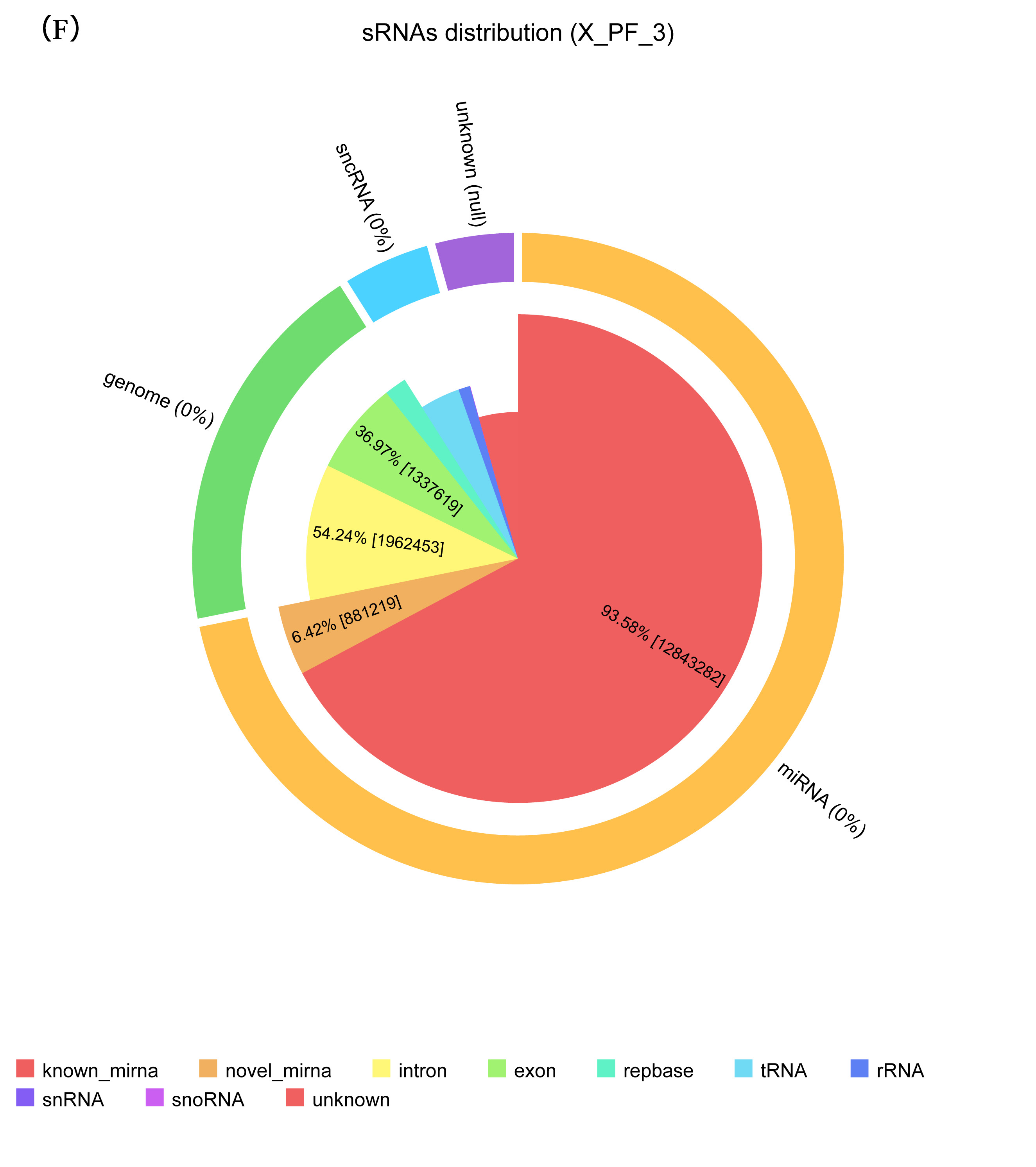

Supplement: Supplementary file 1 [file Data_Sheet_1.zip › 938311_SupMaterial/Data Sheet 1/Supplementary table and figure/Supplementary figure/sRNAs-distribution-(X-PF-3).jpg]
